# Items

**Published:** 1906-11

**Authors:** 


### ITEMS.

THE approach of the holiday season calls to mind the fact that physicians, equally with other persons, are interested in the proper preparation of the table delicacies and luxuries that serve to make for good cheer during the festivities. Among other things two articles are indispensable in making up for these occasions. One of these is Apollinaris Water, which is unrivalled for the table and holds its own as the "Queen" of table waters. The other is "Moet and Chandon White Seal Champagne, vintage of 1900," which is unexcelled by any sparkling wine in the market. It is proper to remark, too, that both "Apollinaris" and "Moet and Chandon" are often of value in the sick room. Whenever sparkling water or wine is indicated these are each at the top of the list in their class.

---

TO CHESS PLAYERS.—The Tri-state Chess Association, an organisation composed of over four hundred players, most of whom reside in the Mississippi Valley, is arranging a correspondence match at chess, the Doctors vs. the Laity. It is desired to have physicians from every section of the United States engaged in this match. Therefore, every chess loving physician is urged to become a consultant in the case. The match will begin early in November; entries will be accepted until January 1, 1907. All who will play are urged to send their names and addresses to the president, with the number of games they will take on. There is no fee attached to the match. Address,

DR. VAN NUYS, President, Lorain, O.

